# Hematocrit-to-Hemoglobin Ratio as a Novel Independent Predictor for In-Hospital Mortality and Delayed Cerebral Ischemia in Critically Ill Patients with Aneurysmal Subarachnoid Hemorrhage Requiring Neurosurgical or Endovascular Treatment: A Retrospective Analysis

**DOI:** 10.1007/s12028-025-02395-x

**Published:** 2025-10-24

**Authors:** Emanuel Moisa, Georgeana Tuculeanu, Liliana Mirea, Claudia Mihail, Stefanita Dima, Liviu Ioan Serban, Dan Corneci, Federico Bilotta, Silvius Ioan Negoita

**Affiliations:** 1 Department of Anesthesiology and Critical Care, Carol Davila University of Medicine and Pharmacy, Dionisie Lupu Street, No. 37, 020021 Bucharest, Romania; 2 Department of Anesthesiology and Critical Care, Elias Emergency University Hospital of Bucharest, Mărăsti Blvd., No. 17, 011461 Bucharest, Romania; 3 Department of Anesthesiology and Critical Care, Clinical Emergency Hospital of Bucharest, Floreasca Avenue, No. 8, 014461 Bucharest, Romania; 4 Department of Interventional Radiology, Elias Emergency University Hospital of Bucharest, Mărăsti Blvd., No. 17, 011461 Bucharest, Romania; 5 Department of Anesthesiology and Critical Care, Dr. Carol Davila Central Military, Emergency University Hospital, Plevnei Avenue, No. 134, 010825 Bucharest, Romania; 6 Department of Anesthesiology and Critical Care, University Tor Vergata of Rome, Via Cracovia, 50, 00133 Rome, Italy

**Keywords:** Aneurysmal subarachnoid hemorrhage, Delayed cerebral ischemia, Blood viscosity, ICU, Hematocrit-to-hemoglobin ratio, Rheology

## Abstract

**Background:**

Substantial research has been dedicated to new prognostication tools in aneurysmal subarachnoid hemorrhage (aSAH), with a recent focus on laboratory parameters. Our study investigates the predictive value of a new hematological index, the hematocrit-to-hemoglobin (Ht-to-Hb) ratio, for mortality and delayed cerebral ischemia (DCI).

**Methods:**

This is a retrospective, single-center, observational study on 78 adult critically ill patients with aSAH. We collected data from the electronic and written records, including demographic and clinical data, therapeutic measures, and intensive care unit and hospital length of stay. The primary outcome was in-hospital mortality, whereas the secondary outcome was DCI development. After descriptive analysis was performed, the Ht-to-Hb ratio was tested as a predictor for the primary and secondary outcomes. Firstly, we conducted a receiver operating characteristic analysis, and cutoff values were identified using the Youden index. Further, in-hospital mortality and DCI probability were evaluated using the log rank test. Cox proportional hazards regression was conducted to test the independent predictive value of the Ht-to-Hb ratio for the aforementioned outcomes.

**Results:**

Mortality during hospitalization was 25.54%, whereas DCI frequency was 42.3%. The Ht-to-Hb ratio had areas under the receiver operating characteristic curve for death prediction and DCI of 0.85 and 0.734, respectively. Values greater than the cutoff value for mortality, 3.069, were independently associated with the primary outcome in the multivariable analysis (hazard ratio [HR] 4.64, 95% confidence interval [CI] 1.08–19.98, *p* = 0.039). For DCI, the cutoff value identified was 3.007. Ht-to-Hb ratios > 3.007 were an independent risk factor for DCI in the multivariable analysis (HR 3.86, 95% CI 1.43–10.4, *p* = 0.008).

**Conclusions:**

The present study proposes a new prognostic index for mortality and DCI in aSAH: the Ht-to-Hb ratio. This marker could act as a surrogate for blood viscosity, uncovering the importance of blood rheology in aSAH pathogenesis.

**Supplementary Information:**

The online version contains supplementary material available at 10.1007/s12028-025-02395-x.

## Introduction

Research on aneurysmal subarachnoid hemorrhage (aSAH) during the last 30 years has provided an increasing number of evidence-based recommendations listed in societies’ guidelines, concurrently with a significant decline in the level of evidence assigned [1]. Important topics, such as outcome prediction and medical management of aSAH, are primarily supported by level B evidence in current guidelines, stressing the urgency of validated prognostication tools and therapeutic strategies [2].

Currently, prognostication in patients with aSAH mainly relies on a combination of clinical (i.e., Hunt and Hess [HH] grade, World Federation of Neurosurgical Societies [WFNS] grade) and radiological (i.e., modified Fisher [mFisher]) scales [2]. Several authors have systematically reviewed evidence on demographic, clinical, and laboratory parameters as potential risk factors for adverse outcomes in aSAH [3–5], although none has emerged thus far as a reliable tool in clinical practice.

Delayed cerebral ischemia (DCI) represents a frequent complication of aSAH that can impact functional outcome to varying degrees. Besides large vessel vasospasm, DCI is also explained by early brain injury, autoregulatory dysfunction, neuroinflammation, cortical spreading depolarizations, and microthrombosis [6, 7]. In light of these mechanistic findings, increasing interest is directed toward blood components [8, 9] and inflammatory [10] and coagulation-related prognostic markers [11].

Amid this plethora of findings, the bedside clinician still lacks practical assets to predict and impact patients’ prognosis because most markers are either poorly validated, not easily available, or nonmodifiable. Subsequently, an extensively researched issue across the years, i.e., blood products and fluid management in aSAH, has experienced a new wave of scrutiny. Anemia negatively impacts long-term neurological outcomes and survival [12], but studies on the effects of red blood cell transfusions have provided conflicting results [13–15]. Novel insights focus on hemoglobin (Hb) and hematocrit (Ht) drifts, in conjunction with fluid therapy, as more accurate and manageable risk factors [16–20]. Some physiological basis is provided by findings on blood viscosity (BV) as a risk factor for DCI development [21].

Thereby, our study aims to describe a new predictive marker for functional outcomes and mortality in aSAH, the hematocrit-to-hemoglobin (Ht-to-Hb) ratio, which could better uncover the crosstalk among blood rheology, fluid therapy, and microcirculatory dysfunction involved in aSAH pathogenesis.

## Methods

### Study Design and Population

This is a retrospective, single-center, observational study in which we included adult (≥ 18 years old) critically ill patients with aSAH treated at Elias Emergency University Hospital (an academic teaching hospital) in Bucharest, Romania. The study period was July 2017 to December 2024 and was approved by the local ethics committee (study number 10108-2/01.08.2023). The inclusion criteria were adult critically ill patients with aSAH treated with surgical clipping or endovascular procedures. The exclusion criteria were as follows: (1) non-aSAH, (2) patients not admitted in the intensive care unit (ICU), (3) conservative treatment (due to patient or relative refusal, delayed presentation, inaccessible or complex anatomy, prolonged prehospital cardiopulmonary resuscitation times, multiorgan failure), (4) patients with end-stage oncological or organ diseases (heart failure, respiratory failure, cirrhosis, chronic kidney disease), (5) patients transferred to another center for specialty treatment, (6) patients transferred from a different center (> 48 h from aneurysm rupture), (7) patients resuscitated from an in-hospital cardiac arrest for > 10 min, and (8) patients in whom death from cardiac arrest or brain death was declared within 48 h after admission. Patients not admitted to the ICU (*n* = 11) were instead admitted to a high-dependency unit and were not listed as a separate category in the population flowchart because they met at least one other exclusion criterion (good HH grade, aSAH with delayed presentation, transferred to another hospital, and patient or relative refusal). All patients were treated according to the available guidelines at that moment [2, 22] and individualized at the physician’s discretion. Withdrawal of life support was not performed in any patient because this decision is not regulated in the Romanian health care system legislation.

### Data Collection

We collected data from the electronic and written records, which included the following: demographic data (age, sex, body mass index), associated diseases (cardiovascular, respiratory, hepatic and oncological diseases, diabetes mellitus), neurological status at ICU admission, and aneurysm treatment modality. Moreover, we collected imagistic data regarding vascular territory for aneurysm rupture, aSAH localization and thickness (mFisher), severity scores (HH scale, Glasgow Coma Scale [GCS], WFNS, modified WFNS [mWFNS], and VASOGRADE), laboratory parameters at ICU admission (Hb; Ht; absolute count of neutrophils, lymphocytes, and platelets; blood glucose level; plasma sodium and potassium levels; serum creatinine level), medical treatment data (antiseizure drugs, vasospasm prophylaxis, antiplatelet and anticoagulant drugs, steroids, statin, and the use of blood products), mechanical ventilation need, and ICU and hospital length of stay (LOS). The following neurological complications were taken into consideration: rebleeding, hydrocephalus, vasospasm, DCI, syndrome of inappropriate antidiuretic hormone secretion, cerebral salt wasting syndrome (CSWS), central diabetes insipidus, seizures, and brain death.

### Definitions and Outcomes

The primary outcome was in-hospital mortality, whereas the secondary outcomes were (1) DCI development during hospital stay and (2) 30-, 60- and 90-day mortality. The following severity scores were retrieved from the patient’s records: GCS score, HH grade, WFNS score, and mFisher score. The mFisher score was graded as published [23]. Two scores were calculated retrospectively based on the original reports: the modified WFNS [24] and the VASOGRADE score [25]. Vasospasm was taken into consideration only if it was reported in the patient’s records and was diagnosed based on angiography or transcranial Doppler. DCI was defined as “cerebral infarction identified on CT or MRI or proven at autopsy, after exclusion of procedure-related infarctions” or as “the occurrence of focal neurological impairment…or a decrease of at least 2 points on the Glasgow Coma Scale,” with or without radiological findings, that “cannot be attributed to other causes by means of clinical assessment, CT or MRI scanning of the brain, and appropriate laboratory studies” [26]. The DCI diagnosis was made by the attending intensivist physician and at least a neurologist, neurosurgeon, or neurointerventional radiologist using clinical examination, laboratory findings, and either a computed tomography (CT) scan or a magnetic resonance imaging (MRI) scan. When relevant, cerebral angiography, transcranial Doppler, and electroencephalography (EEG) were considered as complementary studies. Treatment of DCI included supportive measures (optimization of blood pressure and volemia), enteral nimodipine, and, in cases in which DCI was due to severe and refractory vasospasm, intraarterial vasodilators and/or cerebral angioplasty. The Hb transfusion threshold for packed red blood cells (PRBCs) was set at 9 g/dL. For the calculation of the Ht-to-Hb ratio, several conditions were applied: at least two hemograms were performed, on different days, during the first 3 or 5 days depending on the studied outcome. The ratio was calculated using the Ht (percentage) and Hb (g/dL) level from the same blood sample collected that day. For patients with more than one hemogram performed in one day, the worst Ht-to-Hb ratio was taken into consideration for that day. The worst Ht-to-Hb ratio was defined as the highest value obtained during the first 5 or 3 days depending on the studied outcome. Our decision to use the highest value was based on the hypothesis that the Ht-to-Hb ratio is a viscosity surrogate and that higher values are associated with worse outcomes, as previously reported [21]. The values of Ht and Hb reported by our acid-base balance analyzers were not taken into consideration given that Hb is measured indirectly based on plasma conductance. All hemograms were performed on the same hematology analyzer (XN-series, Sysmex, Kobe, Japan).

### Statistical Analysis

Variables introduced in the analysis were tested for normality of data distribution using the Kolmogorov–Smirnov test. Continuous variables following a normal distribution were reported as mean ± SD, whereas those nonnormally distributed were expressed as median and interquartile range (Q1, Q3). Absolute (number) and relative (percentage) frequencies were used for categorical data. The χ^2^ test was conducted to compare two categorical variables, whereas *t*-test and Mann–Whitney *U*-test were used to compare means or medians from two independent groups. After descriptive analysis was performed, the Ht-to-Hb ratio was tested as a predictor for the primary and secondary outcomes. First, we conducted a receiver operating characteristic (ROC) analysis, and the value for the area under the ROC curve (AUROC) was reported. Furthermore, a cutoff value was identified using the Youden index. Sensitivity, specificity, and positive and negative likelihood ratios (+ LR, − LR) were reported with a 95% confidence interval (CI) for the cutoff values. Moreover, the AUROCs of other known predictive variables were reported and compared with the AUROC for the Ht-to-Hb ratio using the DeLong method [27]. Subsequently, Kaplan–Meier curves were plotted for each outcome studied, and the cutoff value of the Ht-to-Hb ratio (categorical variable) was introduced as an independent factor in the analysis. Cox proportional hazards regression was conducted to test the independent predictive value of the Ht-to-Hb ratio for death and the development of DCI. The Enter method was used for factors introduced in the regression. Thus, univariable analysis was performed for each factor considered in the Cox regression, and multivariable analysis was further performed to identify predictors independently associated with the studied outcome. Even if a factor was not significantly associated with the outcome in the univariable analysis, we introduced it in the multivariable analysis based on previous reports and clinical reasoning. Full models were reported for each outcome and included the Harrell C-index, β value, SE, and hazard ratio (HR) with 95% CI. A *p* value of < 0.05 was considered statistically significant.

Our results were reported with respect to the TRIPOD + AI statement (Transparent Reporting of a Multivariable Prediction Model for Individual Prognosis or Diagnosis Checklist + Artificial Intelligence) [28]. The TRIPOD checklist is available in Supplementary Material 1. For this study, IBM Statistical Package for Social Sciences (SPSS) for Windows version 26.0 (IBM Corp., Armonk, NY) and MedCalc Software version 20.106 (Ostend, Belgium) were used.

## Results

**Fig. 1:**
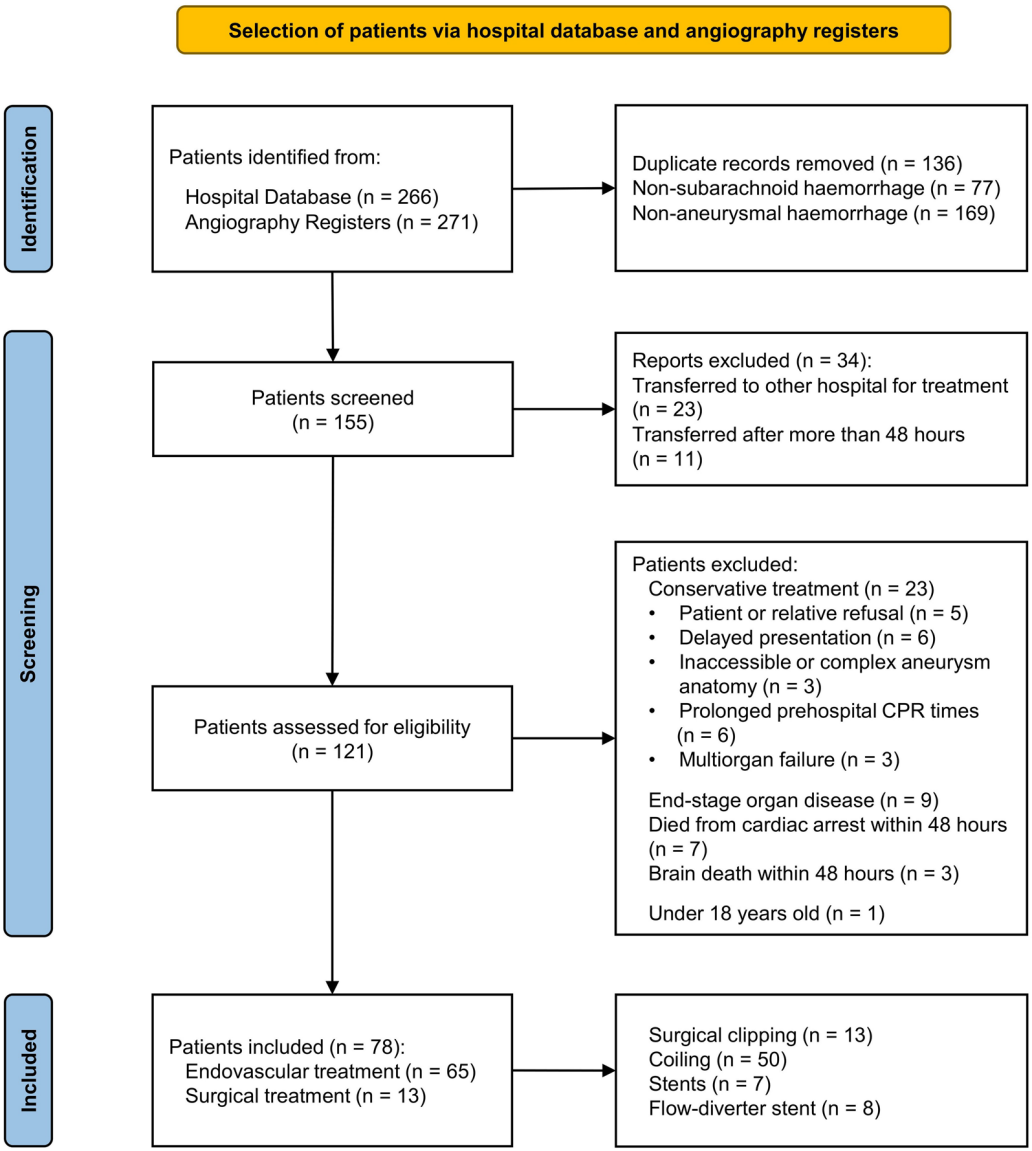
Flowchart of the study population. CPR cardiopulmonary resuscitation

Seventy-eight patients were included in the final analysis (Fig. 1). Results are presented with respect to the primary and secondary outcomes. Characteristics of the studied population in relation to primary and secondary outcomes are summarized in Table 1 and Supplementary Material 2.

**Table 1:** Characteristics of the study population

	Total sample (*N* = 78)	Survivors (*n* = 58)	Nonsurvivors (*n* = 20)	*p* value	DCI group (*n* = 33)	Non-DCI group (*n* = 45)	*p* value
Demographics							
Age, years, mean ± SD	51.1 ± 12.45	50.19 ± 12.73	53.75 ± 11.49	0.25	51.88 ± 11.84	50.53 ± 12.99	0.8
Sex, female, *n* (%)	49/78 (62.8%)	38/58 (65.5%)	11/20 (55%)	0.4	25/33 (75.8%)	24/45 (53.3%)	0.04
CCI, median (IQR)	1 (0–2)	1 (0–2)	1 (0–2)	0.68	1 (0—2)	1 (0—2)	0.78
Hypertension, *n* (%)	50/78 (64.1%)	40/58 (69%)	10/20 (50%)	0.13	19/33 (57.6%)	31/45 (68.9%)	0.3
Dyslipidemia, *n* (%)	33/78 (42.3%)	25/58 (43.1%)	8/20 (40%)	0.80	13/33 (39.4%)	20/45 (44.4%)	0.65
Diabetes mellitus, *n* (%)	5/78 (6.4%)	4/58 (6.9%)	1/20 (5%)	0.76	3/33 (9.1%)	2/45 (4.4%)	0.41
Obesity, *n* (%)	12/78 (15.4%)	9/58 (15.5%)	3/20 (15%)	0.95	4/33 (12.1%)	8/45 (17.8%)	0.49
Smoking, *n* (%)	24/78 (30.8%)	21/58 (36.2%)	3/20 (15%)	0.08	9/33 (27.3%)	15/45 (33.3%)	0.57
Clinical status at admission							
GCS, median (IQR)	14 (8.75–15)	15 (14–15)	6.5 (3.25–11)	< 0.001	13 (5–14.5)	15 (13.5 – 15)	< 0.01
Hunt and Hess grade, *n* (%)							
I	12/78 (15.4%)	12/58 (20.7%)	0/20 (0%)	< 0.001	4/33 (12.1%)	8/45 (17.8%)	0.05
II	30/78 (38.5%)	28/58 (48.3%)	2/20 (10%)		9/33 (27.3%)	21/45 (46.7%)	
III	14/78 (17.9%)	10/58 (17.2%)	4/20 (20%)		7/33 (21.2%)	7/45 (15.6%)	
IV	9/78 (11.5%)	5/58 (8.6%)	4/20 (20%)		3/33 (9.1%)	6/45 (13.3%)	
V	13/78 (16.7%)	3/58 (5.2%)	10/20 (50%)		10/33 (30.3%)	3/45 (6.7%)	
Hunt and Hess grade ≥ 4					14/33 (42.4%)	9/33 (20.0%)	0.03
World Federation of Neurological Societies scale, *n* (%)							
1	36/78 (46.2%)	35/58 (60.3%)	1/20 (5%)	< 0.001	9/33 (27.3%)	27/45 (60.0%)	0.03
2	8/78 (10.3%)	5/58 (8.6%)	3/20 (15%)		4/33 (12.1%)	4/45 (8.9%)	
3	12/78 (15.4%)	10/58 (17.2%)	2/20 (10%)		7/33 (21.2%)	5/45 (11.1%)	
4	6/78 (7.7%)	3/58 (5.2%)	3/20 (15%)		2/33 (6.1%)	4/45 (8.9%)	
5	16/78 (20.5%)	5/58 (8.6%)	11/20 (55%)		11/33 (33.3%)	5/45 (11.1%)	
Modified World Federation of Neurological Societies scale, *n* (%)							
GCS 15	32/78 (41%)	31/58 (53.4%)	1/20 (5%)	< 0.001	8/33 (24.2%)	24/45 (53.3%)	0.08
GCS 14	16/78 (20.5%)	14/58 (24.1%)	2/20 (10%)		8/33 (24.2%)	8/45 (17.8%)	
GCS 13	6/78 (7.7%)	5/58 (8.6%)	1/20 (5%)		3/33 (9.1%)	3/45 (6.7%)	
GCS 7–12	9/78 (11.5%)	3/58 (5.2%)	6/20 (30%)		4/33 (12.1%)	5/45 (11.1%)	
GCS 3–6	15/78 (19.2%)	5/58 (8.6%)	10/20 (50%)		10/33 (30.3%)	5/45 (11.1%)	
Modified Fisher score, *n* (%)							
1	11/78 (14.1%)	11/58 (19%)	0/20 (0%)	< 0.001	4/33 (12.1%)	7/45 (15.6%)	0.81
2	13/78 (16.7%)	12/58 (20.7%)	1/20 (5%)		5/33 (15.2%)	8/45 (17.8%)	
3	14/78 (17.9%)	13/58 (22.4%)	1/20 (5%)		5/33 (15.2%)	9/45 (20.0%)	
4	40/78 (51.3%)	22/58 (37.9%)	18/20 (90%)		19/33 (57.6%)	21/45 (46.7%)	
VASOGRADE score, *n* (%)							
Green	19/78 (24.4%)	18/58 (31%)	1/20 (5%)	< 0.001	7/33 (21.2%)	12/45 (26.7%)	0.17
Yellow	37/78 (47.4%)	32/58 (55.2%)	5/20 (25%)		13/33 (39.4%)	24/45 (53.3%)	
Red	22/78 (28.2%)	8/58 (13.8%)	14/20 (70%)		13/33 (39.4%)	9/45 (20.0%)	
Aneurysm localization, *n* (%)							
Anterior circulation	57/78 (73.1%)	42/58 (72.4%)	15/20 (75%)	0.82	24/33 (72.7%)	33/45 (73.3%)	0.95
Posterior circulation	21/78 (26.9%)	16/58 (27.6%)	5/20 (25%)		9/33 (27.3%)	12/45 (26.7%)	
Laboratory parameters at admission							
Neutrophil count, × 10^9^/L, mean ± SD	10.91 ± 4.53	9.61 ± 3.67	14.64 ± 4.8	< 0.001	11.91 ± 4.96	10.17 ± 4.09	0.11
Lymphocytes count, × 10^9^/L, median (IQR)	1.4 (1–2.09)	1.49 (1.05–2.09)	1.26 (0.76–2.16)	0.25	1.34 (1–2.03)	1.45 (1.00–2.43)	0.58
Platelet count, × 10^9^/L, mean ± SD	249.08 ± 71.54	248.29 ± 72.27	251.35 ± 71.18	0.87	245.56 ± 69.98	251.65 ± 73.34	0.98
INR, median (IQR)	1.04 (1–1.1)	1.04 (1–1.1)	1.04 (0.98–1.08)	0.9	1.04 (1.00 – 1.08)	1.04 (0.98–1.11)	0.87
Blood glucose, mg/dL, median (IQR)	136.5 (111–171)	123.5 (102–149)	192 (160–231)	< 0.001	155 (122–186)	124 (107–162)	0.09
Na, mean ± SD	138.54 ± 4.31	138.37 ± 3.94	139.03 ± 5.3	0.62	138.06 ± 5.59	138.84 ± 3.08	0.31
K, median (IQR)	3.9 (3.5–4.1)	3.88 (3.6–4.1)	3.9 (3.23–4.15)	0.48	3.76 (3.45–4.15)	3.90 (3.55–4.08)	0.36
Creatinine, mean ± SD	0.65 ± 0.18	0.65 ± 0.19	0.67 ± 0.18	0.65	0.61 ± 0.16	0.68 ± 0.19	0.13
Hemoglobin, g/dL, mean ± SD	13.54 ± 1.64	13.37 ± 1.55	14.05 ± 1.81	0.14	13.18 ± 1.95	13.81 ± 1.32	0.25
Hematocrit, %, mean ± SD	39.79 ± 4.71	39.14 ± 4.27	41.67 ± 5.49	0.07	39.03 ± 5.67	40.35 ± 3.84	0.06
Ht-to-Hb ratio, mean ± SD	2.94 ± 0.11	2.93 ± 0.11	2.97 ± 0.13	0.32	2.96 ± 0.11	2.96 ± 0.11	0.11
Aneurysm treatment modality, *n* (%)							
Surgical clipping	13/78 (16.7%)	10/58 (17.2%)	3/20 (15%)	0.75	4/33 (9.1%)	9/45 (20.0%)	0.69
Coils	50/78 (64.1%)	38/58 (65.5%)	12/20 (60%)		22/33 (66.7%)	28/45 (62.2%)	
Stent	7/78 (9%)	4/58 (6.9%)	3/20 (15%)		4/33 (12.1%)	3/45 (6.7%)	
Flow-diverter stent	8/78 (10.3%)	6/58 (10.3%)	2/20 (10%)		3/33 (9.1%)	5/45 (11.1%)	
Neurological complications, *n* (%)							
Rebleeding	9/78 (11.5%)	2/58 (3.4%)	7/20 (35%)	< 0.001	5/33 (15.2%)	4/45 (8.9%)	0.39
Hydrocephalus	8/78 (10.3%)	4/58 (6.9%)	4/20 (20%)	0.09	4/33 (8.9%)	4/45 (8.9%)	0.64
Vasospasm	37/78 (47.4%)	26/58 (44.8%)	11 (55%)	0.43	25/33 (75.8%)	12/45 (26.7%)	< 0.001
DCI	33/78 (42.3%)	17/58 (29.3%)	16/20 (80%)	< 0.001	–	–	–
SIADH	3/78 (3.8%)	2/58 (3.4%)	1/20 (5%)	0.76	1/33 (3.0%)	2/45 (4.4%)	0.75
CSWS	4/78 (5.1%)	3/58 (5.2%)	1/20 (5%)	0.98	4/33 (12.1%)	0/45 (0.0%)	0.02
Diabetes insipidus	9/78 (11.5%)	0 (0%)	9/20 (45%)	< 0.001	7/33 (21.2%)	2/45 (4.4%)	0.02
Seizures	16/78 (20.5%)	10/58 (17.2%)	6/20 (30%)	0.22	8/33 (24.2%)	8/45 (17.8%)	0.48
Brain death	2/78 (2.6%)				2/33 (6.1%)	0/45 (0.0%)	0.09
Outcomes							
Mechanical ventilation, *n* (%)	34/78 (43.6%)	15/58 (25.9%)	19/20 (95%)	< 0.001	20/33 (60.6%)	14/45 (31.3%)	< 0.01
ICU LOS, days, median (IQR)	4 (2–10)	3 (1–7)	10 (6–16)	< 0.001	9 (4–15)	3 (1–6)	< 0.001
Hospital LOS, days, median (IQR)	15 (10–21)	11.5 (11–21)	12 (6–19)	0.14	17 (11–27)	14 (10–19)	0.07

*CCI* Charlson Comorbidity Index, *CSWS* cerebral salt wasting syndrome, *DCI* delayed cerebral ischemia, *GCS* Glasgow Coma Scale, *Ht-to-Hb* hematocrit-to-hemoglobin, *ICU* intensive care unit, *INR* international normalized ratio, *IQR* interquartile range, *K* potassium, *LOS* length of stay, *Na* sodium, *SIADH* syndrome of inappropriate antidiuretic hormone secretion

*p* < 0.001

### Characteristics of the Studied Population in Relation to Mortality

The survival rate was 74.35% (58 of 78). The mean age was 51.1 (± 12.45) years old, and 62.8% were female, with no difference between studied groups. Cardiovascular diseases were among the most frequent (64.1%), followed by dyslipidemia (42.3%) and obesity (15.4%). Other diseases were infrequent (respiratory 2 of 78, oncological 1 of 78, cirrhosis 1 of 78), and no difference was observed between survivors and nonsurvivors for the associated diseases, smoking status, or the Charlson Comorbidity Index (*p* > 0.05). Subarachnoid hemorrhage was secondary to aneurysm rupture in the anterior circulation in 73% (57 of 78) of cases, but no relation with mortality was observed. Nonsurvivors had significantly lower GCS scores (*p* < 0.001) and higher HH, WFNS, mWFNS, mFisher, and VASOGRADE scores (*p* < 0.001). Most of the patients received endovascular intervention, with only 16% of patients being subjected to neurosurgical clipping. Patients requiring both endovascular and neurosurgical management, as well as patients in need of reintervention, represented a small percentage but had higher mortality rates (*p* < 0.001). In patients managed through interventional procedures, antiplatelet therapy was associated with better survival (*p* < 0.001), as well as the use of steroids (*p* < 0.001). Other medical treatments did not influence mortality. Rebleeding (*p* < 0.001), DCI (*p* < 0.001), and development of diabetes insipidus (*p* < 0.001) were associated with higher mortality. Of nine patients with rebleeding, this complication occurred early (first 24 h) in four patients: at 48 h for two patients, with the remaining at days 5, 7, and 15. External ventricular drains were used in five of nine patients with hydrocephalus, with no difference in mortality rate (50% vs. 75%, *p* = 0.47). Nonsurvivors required invasive mechanical ventilation more frequently (*p* < 0.001) and had a significantly higher median ICU LOS (*p* < 0.001), but no difference was observed for hospital LOS between the studied groups. Neutrophilia (*p* < 0.001) and hyperglycemia (*p* < 0.001) at ICU admission were associated with lower survival rates. Of 58 survivors, 12 (20.3%) were discharged to a long-term care facility, whereas the remaining 40 (79.3%) were discharged to home.

### Characteristics of the Studied Population in Relation to DCI Development

DCI developed in 33 patients (42.3%) at a median of 6 (IQR 5–9) days and was more frequent in female patients (*p* = 0.043). No association with DCI was observed for other demographic factors, associated diseases, aneurysm rupture location, endovascular, surgical or medical treatment, and laboratory parameters. Lower GCS scores (*p* < 0.01), an HH score ≥ 4 (*p* = 0.032), and a lower WFNS score (*p* = 0.031) were significantly correlated with DCI development. No association with DCI was observed for mWFNS, mFisher, and VASOGRADE scores (*p* > 0.05). Patients with DCI presented a higher proportion of vasospasm (*p* < 0.001), diabetes insipidus (*p* = 0.02), and CSWS (*p* = 0.02). Seizure frequency did not differ among groups, and EEG was used as a complementary diagnostic study for DCI in ten patients. No difference was observed in DCI rates between patients with and without a ventriculostomy drain for hydrocephalus (75% vs. 50%, *p* = 0.47). Furthermore, patients with DCI received more PRBCs (*p* = 0.031), but no difference between the mean values of the Ht-to-Hb ratio was noted at any given time in relation to PRBC transfusion (*p* > 0.05). Lastly, patients had a longer ICU LOS (*p* < 0.001), but no difference was observed regarding hospital LOS in this group.

### ROC Analysis for the Ht-to-Hb Ratio as a Predictor for the Studied Outcomes

#### ROC Analysis for in-Hospital Mortality

**Fig. 2:**
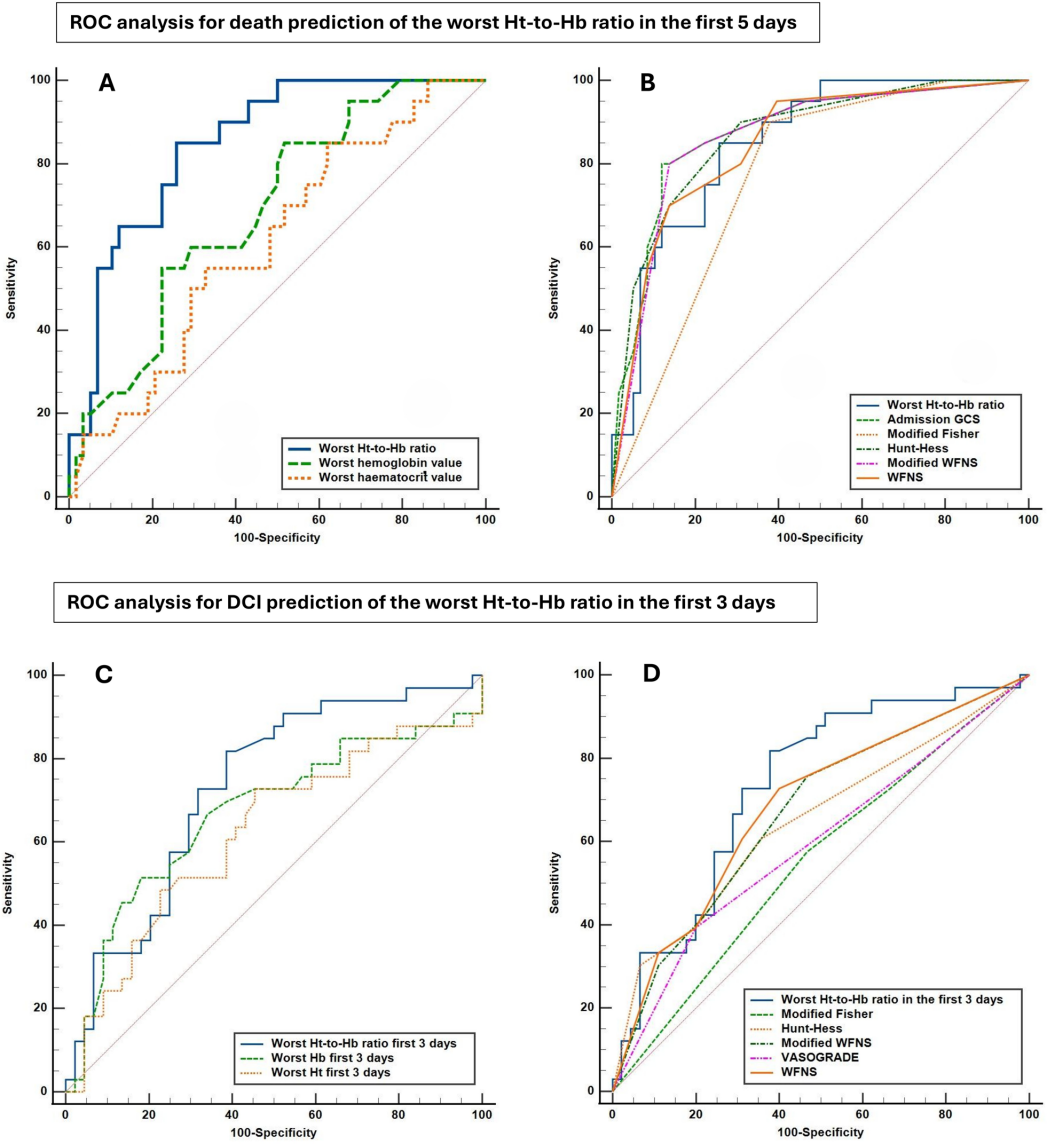
Receiver operating characteristic (ROC) analysis for the prediction of the studied outcomes. Primary outcome: in-hospital mortality (**a** and **b**). **a** The worst hematocrit-to-hemoglobin (Ht-to-Hb) ratio in the first 5 days is compared against Ht and Hb alone. **b** The worst Ht-to-Hb ratio in the first 5 days is compared against the following scores at intensive care unit admission: Hunt–Hess grade, Glasgow Coma Scale (GCS), World Federation of Neurosurgical Societies (WFNS) and its modified version (mWFNS), modified Fisher score and VASOGRADE (not shown here but reported in Table 2). Secondary outcome: delayed cerebral ischemia (DCI) prediction (**c** and **d**). **c** The worst Ht-to-Hb ratio in the first 3 days is compared against Ht and Hb alone. **d** The worst Ht-to-Hb ratio in the first 3 days is compared against the same scores as in panel **b** (VASOGRADE shown here, but GCS reported only in Table 3)

A total of 308 hemograms were performed in the first 5 days of ICU admission in all patients. Higher Ht-to-Hb ratio values were observed in nonsurvivors (3.15 ± 0.08 vs. 3.01 ± 0.1, *p* < 0.001). The worst value of the Ht-to-Hb ratio in the first 5 days was included in the ROC analysis. The AUROC for death prediction was 0.85 (95% CI 0.75–0.92, *p* < 0.001) and was significantly higher compared to the AUROCs of Ht (*p* < 0.01) and Hb (*p* < 0.05) alone (Fig. 2a). Based on the Youden index, a cutoff value of > 3.069 was identified, and sensitivity, specificity, + LR, and − LR are reported in Table 2. A proportion of 42.3% of patients had an Ht-to-Hb ratio > 3.069, and 85% of them died. No significant difference was observed for the AUROCs of GCS, HH, WFNS, mWFNS, mFisher, and VASOGRADE scores in comparison to the AUROC for the Ht-to-Hb ratio (Table 2, Fig. 2b). The AUROC values for 30-, 60- and 90-day mortality are reported in Supplementary Material 4.

**Table 2:** Results derived from the receiver operating characteristic analysis for in-hospital mortality prediction

The predictive power for death of the Ht-to-Hb ratio						
Death prediction	AUC (95% CI)	Youden index (95% CI)	Sensitivity (95% CI)	Specificity (95% CI)	+ LR (95% CI)	–LR (95% CI)
Ht-to-Hb ratio cutoff: > 3.069 (3.02–3.14)	0.85 (0.75–0.92)	0.59 (0.36–0.71)	85% (62.1–96.8)	74.14% (61–84.7)	3.29 (2.05–5.27)	0.20 (0.07–0.58)
Comparison	Hematocrit AUC: 0.61, 95% CI 0.49–0.72	Hemoglobin AUC: 0.69, 95% CI 0.58–0.79				
Ht-to-Hb ratio	AUC difference: 0.24, *p* = 0.009	AUC difference: 0.16, *p* = 0.048				

Comparison between the AUC of the Ht-to-Hb ratio with other predictive scores							
Death	Ht-to-Hb	GCS	Hunt–Hess	WFNS	mWFNS	mFisher	VASOGRADE
AUC (95% CI)	0.85 (0.75–0.92)	0.87 (0.78–0.94)	0.87 (0.77–0.93)	0.85 (0.75–0.92)	0.87 (0.77–0.93)	0.77 (0.66–0.86)	0.81 (0.7–0.89)
AUC difference (*p* value)		0.02 (0.68)	0.02 (0.75)	0 (0.99)	0.02 (0.79)	0.08 (0.17)	0.04 (0.13)

*AUC* area under the curve, *CI* confidence interval, *GCS* Glasgow Coma Scale, *Ht-to-Hb* hematocrit-to-hemoglobin, *LR* likelihood ratio, *mFisher* modified Fisher score, *mWFNS* modified World Federation of Neurosurgical Societies, *WFNS* World Federation of Neurosurgical Societies

#### ROC Analysis for DCI Development

A total of 200 hemograms were performed in the first 3 days of ICU admission in all patients. Higher Ht-to-Hb ratio values were observed in the DCI group (2.99 ± 0.1 vs. 3.07 ± 0.1). The worst value of the Ht-to-Hb ratio in the first 3 days was included in the ROC analysis. The AUROC for DCI prediction was 0.734 (95% CI 0.62–0.83, *p* < 0.001), but it was not significantly higher compared to the AUROCs of Ht and Hb alone (*p* > 0.05) (Fig. 2c). Based on the Youden index, a cutoff value of > 3.007 was identified. A proportion of 59% of patients had an Ht-to-Hb ratio > 3.007, and 82% of them developed DCI. The AUROC value of the Ht-to-Hb ratio was significantly higher compared to the AUROCs of mFisher and VASOGRADE scores (*p* < 0.05). No significant difference was observed for the AUROCs of HH, WFNS, and mWFNS scores (*p* > 0.05) (Table 3, Fig. 2d).

**Table 3:** Results derived from the receiver operating characteristic analysis for DCI prediction

The predictive power for DCI of the Ht-to-Hb ratio						
DCI	AUC (95% CI)	Youden index (95% CI)	Sensitivity (95% CI)	Specificity (95% CI)	+ LR (95% CI)	− LR (95% CI)
Ht-to-Hb ratio cutoff: > 3.007 (3–3.06)	0.73 (0.62–0.83) *p* < 0.001	0.44 (0.21–0.58)	81.82 (64.5–93)	62.22 (45.5–75.6)	2.17 (1.44–3.26)	0.29 (0.14–0.62)
Comparison	Hemoglobin AUC: 0.67, 95% CI 0.55–0.77			Hemoglobin AUC: 0.67, 95% CI 0.55–0.77		
Ht-to-Hb ratio	AUC difference: 0.11, *p* = 0.17			AUC difference: 0.06, *p* = 0.39		

Comparison between the AUC of the Ht-to-Hb ratio with other predictive scores							
DCI	Ht-to-Hb	GCS	Hunt–Hess	WFNS	mWFNS	mFisher	VASOGRADE
AUC difference (95% CI)	0.73 (0.62–0.83)	0.68 (0.56–0.77)	0.65 (0.53–0.75)	0.68 (0.57–0.79)	0.67 (0.56–0.77)	0.55 (0.44–0.67)	0.59 (0.48–0.70)
AUC difference (*p* value)		0.05 (0.4)	0.08 (0.21)	0.05 (0.46)	0.06 (0.36)	0.18 (0.012)	0.14 (0.032)

*AUC* area under the curve, *CI* confidence interval, *DCI* delayed cerebral ischemia, *GCS* Glasgow Coma Scale, *Ht-to-Hb* hematocrit-to-hemoglobin, *LR* likelihood ratio, *mFisher* modified Fisher score, *mWFNS* modified World Federation of Neurosurgical Societies, *WFNS* World Federation of Neurosurgical Societies

### Kaplan–Meier and Cox Proportional Hazards Regression for Death Prediction

**Fig. 3:**
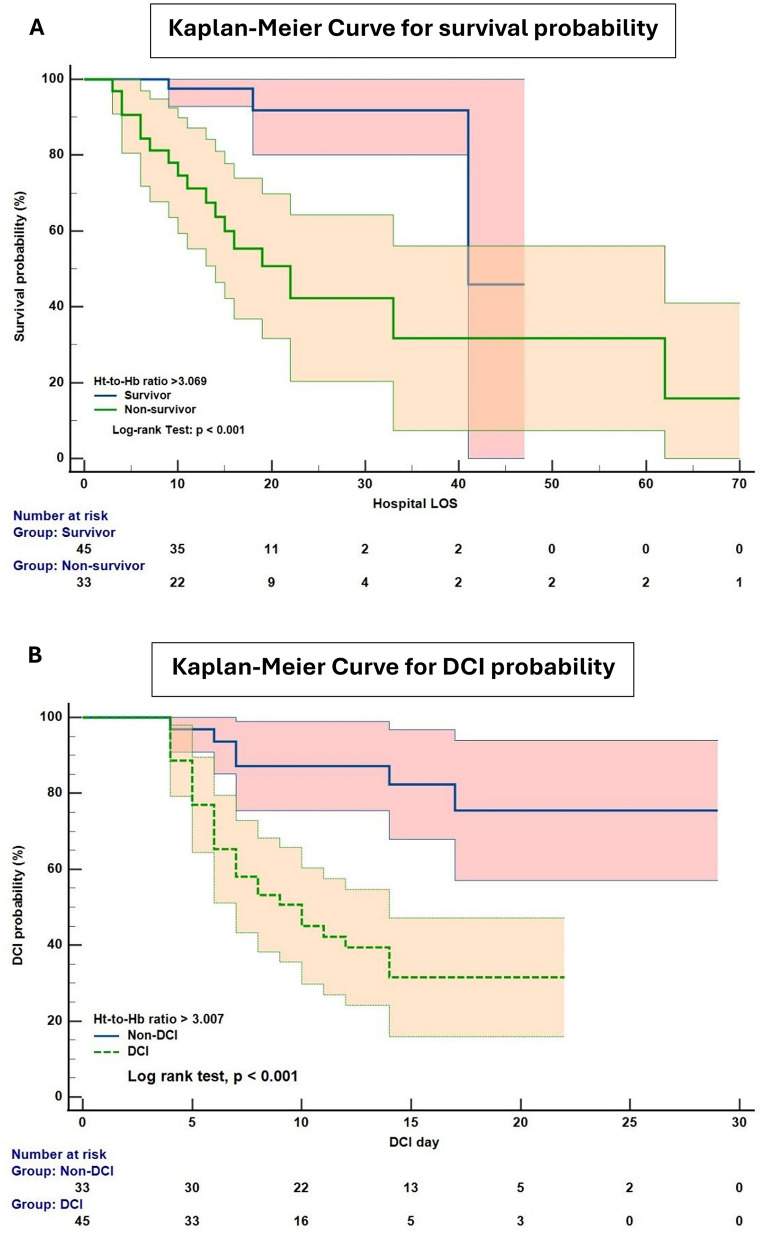
Kaplan–Meier curve for in-hospital survival probability (**a**) and DCI probability (**b**). DCI delayed cerebral ischemia, Ht-to-Hb hematocrit-to-hemoglobin, LOS length of stay

The difference in survival probability between patients with an Ht-to-Hb ratio above or below 3.069 was determined using a log rank test (Fig. 3a). The distribution of mortality was statistically significant between the two groups (χ^2^ = 14.3, *p* < 0.001). The reported HRs were as follows: HR 0.17 (95% CI 0.07–0.43) for Ht-to-Hb ratio ≤ 3.069 and HR 5.9 (95% CI 2.35–14.82) for Ht-to-Hb ratio > 3.069. The same analysis was conducted for 30-, 60- and 90-day mortality and is reported in Supplementary Material 4.

The Ht-to-Hb ratio was further analyzed as an independent predictor for death. The full regression model is reported in Supplementary Material 3. Following multivariable Cox analysis, three predictors remained independently associated with in-hospital mortality: HH score ≥ 4 (HR 3.67, 95% CI 1.15–11.7, *p* = 0.028), neutrophil absolute count (HR 1.16, 95% CI 1.02–1.33, *p* = 0.029), and an Ht-to-Hb ratio > 3.069 (HR 4.64, 95% CI 1.08–19.98, *p* = 0.039).

### Kaplan–Meier and Cox Proportional Hazards Regression for DCI Prediction

To determine whether there was a significant difference in DCI probability between patients with an Ht-to-Hb ratio above or below 3.007, a log rank test was conducted (Fig. 3b). The distribution of DCI occurrence was statistically significant between the two groups (χ^2^ = 15.27, *p* < 0.001). The reported HRs were as follows: HR 0.24 (95% CI 0.12–0.49) for Ht-to-Hb ratio ≤ 3.007 and HR 4.13 (95% CI 2.03–8.42) for Ht-to-Hb ratio > 3.007.

The Ht-to-Hb ratio was adjusted for several confounders regarding DCI development. All factors introduced in the analysis are reported in Supplementary Material 3. Three variables remained independently associated with the secondary outcome: HH score ≥ 4 (HR 2.6, 95% CI 1.13–5.96, *p* = 0.024), vasospasm (HR 4.3, 95% CI 1.64–11.28, *p* = 0.003), and an Ht-to-Hb ratio > 3.007 (HR 3.86, 95% CI 1.43–10.4, *p* = 0.008).

## Discussion

The present retrospective analysis uncovers a new independent predictive marker for in-hospital mortality and DCI development: the Ht-to-Hb ratio. This index effectively predicted death and DCI, with cutoff values remaining independently associated with outcomes in multivariable models. To the best of our knowledge, this is the first study to describe the Ht-to-Hb ratio as a predictor for in-hospital mortality and DCI in patients with aSAH.

The primary outcome of our study, in-hospital mortality, was 25.54%, which is in line with studies focusing on ICU patients treated after 2015 (23–30%) [29, 30]. DCI developed in 42.3% of our patients and was chosen as the secondary outcome since it is the leading cause of functional disability in survivors [31]. Most sources report an incidence of around 30% [32], though inconsistent DCI definitions limit study comparability [33].

Oxygen delivery to cerebral tissue is crucial, but so are BV and cerebral blood flow [34]. Anemia has been frequently associated with unfavorable outcomes [35, 36], although conflicting results are reported [12, 14]. Several findings suggest that aSAH shows a distinct vulnerability to anemia: (1) worse outcomes mainly after securing the aneurysm or during the DCI window, not at admission [12]; (2) Hb and Ht decrements during LOS worsened outcomes [17, 18, 20]; (3) PRBC transfusions improve outcomes in certain subgroups [12]; and (4) the negative impact of anemia diminished when accounting for fluid balance [16]. On the other hand, elevated diastolic BV (DBV) upon admission was correlated with the development of DCI in aSAH [21]. Similar associations of BV with cerebral infarcts were reported in patients with ischemic stroke with small artery occlusions [37]. Therefore, this new marker could serve as a surrogate for elevated BV, enhancing the understanding of blood components’ roles in aSAH pathogenesis. Interestingly, the AUROC for DCI prediction of an Ht-to-Hb ratio > 3.007 was similar to that of DBV (0.73 vs. 0.79) [21]. Moreover, previous studies have correlated the Ht-to-Hb ratio with thrombotic events [38, 39]. A study on patients with pulmonary embolism proposed a cutoff value of 3.08 to predict mortality, which is close to the one we identified, i.e., 3.069 [40].

Elevated BV increases endothelial sheer stress, promoting endothelial dysfunction and subsequent inflammatory and prothrombotic processes [41]. In this regard, statins [42, 43], antiplatelets [44, 45], and anticoagulants [46] have been proposed as prophylaxis for DCI, with promising results. In our cohort, antiplatelet and anticoagulant therapy decreased mortality, whereas DCI rates were not influenced by any of these agents. Furthermore, in our study, steroid therapy positively influenced survival rates, although therapy groups were disproportionate. Their water-retaining and antiinflammatory effects may have played a role. Reduced viscosity was observed with hydrocortisone [47], possibly due to decreased prostaglandin E2 levels [48]. A study on dexamethasone implants in patients with retinal vein thrombosis noted improved hemorheological profiles [49].

Multiple models or scales (e.g., GCS, HH, WFNS, and mWFNS) were developed or validated as prediction tools in patients with aSAH [50–52]. Of note, in our study, the worst Ht-to-Hb ratio in the first 5 days had a similar AUROC value of 0.85 for in-hospital mortality and was not statistically different from HH, WFNS, mWFNS, or GCS scales. Fisher and mFisher scales [53, 54] and VASOGRADE [25, 55] have modest predictive performance for DCI development. This was similar in our analysis, as the mFisher scale and the VASOGRADE were poor discriminants and were inferior to the worst value of the Ht-to-Hb ratio before the DCI window. Recent advances in DCI pathogenesis highlight the potential of blood-based diagnostic or screening tests [4], stressing the importance of our findings.

The Ht-to-Hb ratio predicted mortality, but not DCI, more accurately than Ht or Hb alone. This suggests that increased BV may worsen outcomes in patients with aSAH through mechanisms beyond DCI (e.g., impaired oxygen delivery, microvascular stasis, and thrombotic risk), similar to findings in other critically ill patients [56, 57]. Although DCI remains a key factor in aSAH mortality, including in our cohort, it involves multiple nonrheological mechanisms [6], which may explain the limited predictive value of the Ht-to-Hb ratio.

Overall, our new predictor offers several advantages: it is widely available, mildly invasive, low cost, and, most importantly, modifiable. Further research could explore individualized fluid resuscitation strategies and transfusion thresholds, as these remain key knowledge gaps in management of patients with aSAH. Current data suggests that liberal fluid administration [19] and transfusion do not improve outcomes [14], but using a more evidence-based patient selection strategy (i.e., Ht-to-Hb ratio greater than 3.1) may alter these findings.

Our study has several limitations. This is a retrospective analysis from a single low-volume center, which increases the risk for incomplete data collection, as well as selection and geographical bias. The latter could play a major role in DCI development and functional outcomes [58]. Furthermore, patients with end-stage diseases or treated in a conservative manner were excluded. This framework mirrors many clinical studies on major negative outcomes in patients with aSAH [59], but it may hinder generalizability and underestimate the actual mortality rate [59]. In addition, we did not adjust our findings to patients’ fluid balance and osmolality due to missing data. We acknowledge this limitation because Ht and Hb can vary widely depending on these parameters [60]. Current guidelines consider continuous EEG (cEEG) reasonable for detection of seizures and predicting DCI [2]. Ten patients in our cohort underwent EEG to refine differential diagnosis. Unfortunately, cEEG is not available in our setting, and this is a limitation. Lastly, we did not distinguish between symptomatic DCI and DCI identified by new cerebral infarction per the unified definition [26]. This could explain the higher DCI frequency, and it possibly influenced the AUROC for the Ht-to-Hb ratio. The impact on predictive value requires further study.

## Conclusions

This study introduces the Ht-to-Hb ratio as an independent predictor of in-hospital mortality and DCI development in critically ill patients with aSAH. The ratio showed very good and good discriminative power for predicting in-hospital mortality and DCI, respectively. It performed comparably or better than other severity scales and predictive scores, remaining independently associated with outcomes in multivariable analyses. Although potentially a surrogate for BV, further research is needed to confirm this hypothesis and any causal relation. Due to the retrospective nature of our study, we advise caution in using this index, as not all confounding factors were adjusted for.

## Supplementary Information

Below is the link to the electronic supplementary material.

Supplementary file1 (DOCX 92 KB)

Supplementary file2 (DOCX 22 KB)

Supplementary file3 (DOCX 33 KB)

Supplementary file4 (DOCX 1074 KB)

## Data Availability

The data presented in this study are available on request from the corresponding author.
