# Supplementary material for: Hematocrit-to-Hemoglobin Ratio as a Novel Independent Predictor for In-Hospital Mortality and Delayed Cerebral Ischemia in Critically Ill Patients with Aneurysmal Subarachnoid Hemorrhage Requiring Neurosurgical or Endovascular Treatment: A Retrospective Analysis
Source: Neurocrit Care. 2025 Oct 24;44(2):527–40. doi: 10.1007/s12028-025-02395-x (PMC13053589; doi:10.1007/s12028-025-02395-x)
Supplement: Supplementary file 2 — Supplementary file2 (DOCX 22 KB) [file 12028_2025_2395_MOESM2_ESM.docx]

**Supplementary material 1. Additional results regarding treatment in the study population**

Table S1. Medical treatment and need for reintervention in the study population

|  | **Total Sample (n=78)** | **Survivors (n=58)** | **Non-survivors (n=20)** | **p value** | **DCI Group (n=33)** | **Non-DCI Group (n=45)** | **p value** |
| --- | --- | --- | --- | --- | --- | --- | --- |
|  | | | | | | | |
| Reintervention, n (%) | | | | | | | |
| None | 72/78 (92.3%) | 56/58 (96.6%) | 16/20 (80%) | **0.04** | 27/72 (37.5%) | 45/72 (62.5%) | **0.012** |
| Angiography | 5/78 (6.4%) | 2/58 (3.4%) | 3/20 (15%) |  | 5/5 (100%) | 0/5 (0%) |  |
| Surgery | 1/78 (1/3%) | 0/58 (0%) | 1/20 (5%) |  | 1/1 (100%) | 0/0 (0%) |  |
| **Medical treatment** | | | | |  |  |  |
| **Anticoagulation in endovascular group, n (%)** | | | | |  |  |  |
| None | 10/66 (15.2%) | 3/49 (6.1%) | 7/17 (41.2%) | **<0.001** | 6/29 (20.7%) | 4/37(10.8%) | 0.300 |
| Prophylactic | 34/66 (51.5%) | 30/49 (61.2%) | 4/17 (23.5%) |  | 12/29 (41.4%) | 22/37 (59.5%) |  |
| Therapeutic | 22/66 (33.3%) | 16/49 (32.7%) | 6/17 (35.3%) |  | 11/29 (37.9%) | 11/37 (29.7%) |  |
| **Antiplatelet agents in interventional group, n (%)** | | | | |  |  |  |
| None | 16/66 (24.2%) | 8/49 (16.3%) | 8/17 (47/1%) | **0.039** | 9/29 (13.6%) | 7/37 (18.9%) | 0.492 |
| Single antiplatelet therapy | 29/66 (43.9%) | 24/49 (49%) | 5/17 (17.2%) |  | 11/29 (37.9%) | 18/37 (48.6%) |  |
| Dual antiplatelet therapy | 21/66 (31.8%) | 17/49 (34.7%) | 4/17 (23.5%) |  | 9/29 (31.0%) | 12/37 (32.4%) |  |
| Steroids, n (%) | 66/78 (84.6%) | 54/58 (93.1%) | 12/20 (60%) | **<0.001** | 26/33 (78.8%) | 40/45 (88.9%) | 0.222 |
| **Vasospasm tratement/prevention, n (%)** | | | | | | | |
| None | 6/78 (7.7%) | 4/58 (6.9%) | 2/20 (10%) | 0.240 | 2/33 (6.1%) | 4/45 (8.9%) | 0.599 |
| Nimodipine | 36/78 (46.2%) | 29/58 (50%) | 7/20 (35%) |  | 13/33 (39.4%) | 23/45 (51.1%) |  |
| MgSO4 | 5/78 (6.4%) | 5/58 (8.6%) | 0/20 (0%) |  | 3/33 (9.1%) | 2/45 (4.4%) |  |
| Nimodipine & MgSO4 | 31/78 (39.7%) | 20/58 (34.5%) | 11/20 (55%) |  | 15/33 (45.5%) | 16/45 (35.6%) |  |
| ≥2 antiseizure drugs, n (%) | 15/78 (19.2%) | 10/58 (17.2%) | 5/20 (25%) | 0.448 | 7/33 (21.2%) | 8/45 (17.8%) | 0.704 |
| Phenytoin, n (%) | 14/78 (17.9%) | 9/58 (15.5%) | 5/20 (25%) | 0.341 | 5/33 (15.2%) | 9/45 (20.05) | 0.581 |
| Statin, n (%) | 18/78 (23.1%) | 13/58 (22.5%) | 5/20 (25%) | 0.813 | 11/33 (33.3%) | 7/45 (15.6%) | 0.066 |
| Blood products, n (%) | 13/78 (16.7%) | 7/58 (12.1%) | 6/20 (30%) | 0.064 | 9/33 (27.3%) | 4/45 (8.9%) | **0.031** |
