# Supplementary material for: Hematocrit-to-Hemoglobin Ratio as a Novel Independent Predictor for In-Hospital Mortality and Delayed Cerebral Ischemia in Critically Ill Patients with Aneurysmal Subarachnoid Hemorrhage Requiring Neurosurgical or Endovascular Treatment: A Retrospective Analysis
Source: Neurocrit Care. 2025 Oct 24;44(2):527–40. doi: 10.1007/s12028-025-02395-x (PMC13053589; doi:10.1007/s12028-025-02395-x)
Supplement: Supplementary file 3 — Supplementary file3 (DOCX 33 KB) [file 12028_2025_2395_MOESM3_ESM.docx]

**Supplementary material 3. Full Cox proportional hazards regression models**

1. **Cox proportional hazards regression for in-hospital mortality prediction**

|  | Univariable analysis | | | | | |
| --- | --- | --- | --- | --- | --- | --- |
| Covariate | β | SE | Wald | p | HR | 95% CI |
| Glycaemia > 180 | **1.2272** | **0.4533** | **7.3283** | **0.0068** | **3.4116** | **1.4031 to 8.2953** |
| Hunt-Hess≥4 | **1.9116** | **0.5230** | **13.3595** | **0.0003** | **6.7639** | **2.4267 to 18.8533** |
| Anterior/Posterior | -0.2469 | 0.5225 | 0.2234 | 0.6365 | 0.7812 | 0.2806 to 2.1751 |
| Neutrophils | **0.1561** | **0.04552** | **11.7654** | **0.0006** | **1.1690** | **1.0692 to 1.2781** |
| Treatment | -0.01803 | 0.6372 | 0.000801 | 0.9774 | 0.9821 | 0.2817 to 3.4242 |
| CCI | -0.03330 | 0.1776 | 0.03515 | 0.8513 | 0.9673 | 0.6829 to 1.3700 |
| Gender | 0.9000 | 0.4970 | 3.2787 | 0.0702 | 2.4596 | 0.9285 to 6.5158 |
| Age | 0.00887 | 0.01973 | 0.2022 | 0.6529 | 1.0089 | 0.9706 to 1.0487 |
| Ht-to-Hb>3.069 | **2.0223** | **0.6310** | **10.2731** | **0.0013** | **7.5560** | **2.1938 to 26.0240** |
| Smoking | -0.5628 | 0.6374 | 0.7797 | 0.3772 | 0.5696 | 0.1633 to 1.9867 |
| Multivariable analysis | | | | | | |
| Method: Enter, p for model <0.001, Harrell’s C index for model: 0.836 (0.765 - 0.907) | | | | | | |
| Covariate | β | SE | Wald | p | HR | 95% CI |
| Glycaemia > 180 | 0.3699 | 0.5654 | 0.4280 | 0.5130 | 1.4476 | 0.4779 to 4.3850 |
| Hunt-Hess≥4 | 1.2994 | 0.5920 | 4.8185 | 0.0282 | 3.6671 | 1.1493 to 11.7006 |
| Anterior/Posterior | -0.8182 | 0.6108 | 1.7945 | 0.1804 | 0.4412 | 0.1333 to 1.4608 |
| Neutrophils | 0.1492 | 0.06840 | 4.7564 | 0.0292 | 1.1609 | 1.0152 to 1.3274 |
| Treatment | -0.2865 | 0.8312 | 0.1188 | 0.7303 | 0.7509 | 0.1472 to 3.8294 |
| CCI | 0.1659 | 0.3732 | 0.1976 | 0.6566 | 1.1804 | 0.5681 to 2.4529 |
| Gender | 1.2936 | 0.6612 | 3.8273 | 0.0504 | 3.6460 | 0.9976 to 13.3254 |
| Age | -0.03212 | 0.04353 | 0.5446 | 0.4605 | 0.9684 | 0.8892 to 1.0546 |
| Ht-to-Hb>3.069 | 1.5335 | 0.7456 | 4.2307 | 0.0397 | 4.6346 | 1.0749 to 19.9827 |
| Smoking | -0.1248 | 0.7563 | 0.02721 | 0.8690 | 0.8827 | 0.2005 to 3.8868 |

1. **Cox proportional hazards regression for DCI prediction**

| Univariable analysis | | | | | | |
| --- | --- | --- | --- | --- | --- | --- |
| Covariate | β | SE | Wald | p | HR | 95% CI |
| **Hunt-Hess ≥4** | **0.7674** | **0.3538** | **4.7044** | **0.0301** | **2.1541** | **1.0767 to 4.3096** |
| **Vasospasm** | **1.4037** | **0.4075** | **11.8647** | **0.0006** | **4.0702** | **1.8312 to 9.0470** |
| **Ht-to-Hb >3.007** | **1.5744** | **0.4559** | **11.9255** | **0.0006** | **4.8278** | **1.9755 to 11.7982** |
| Modified Fisher | 0.09385 | 0.1667 | 0.3168 | 0.5735 | 1.0984 | 0.7922 to 1.5229 |
| Gender | -0.7750 | 0.4065 | 3.6340 | 0.0566 | 0.4607 | 0.2077 to 1.0221 |
| Age | 0.002669 | 0.01323 | 0.04072 | 0.8401 | 1.0027 | 0.9770 to 1.0290 |
| Hypertension | -0.4633 | 0.3546 | 1.7068 | 0.1914 | 0.6292 | 0.3140 to 1.2608 |
| Aneurysm:  Anterior/Posterior | 0.1168 | 0.3913 | 0.08909 | 0.7653 | 1.1239 | 0.5219 to 2.4201 |
| Smoking | -0.1497 | 0.3912 | 0.1466 | 0.7018 | 0.8609 | 0.3999 to 1.8532 |
| Multivariable analysis | | | | | | |
| Method: Enter, p for model <0.001, Harrell’s C-index = 0.767 (0.705 – 0.829) | | | | | | |
| Covariate | β | SE | Wald | p | HR | 95% CI |
| **Hunt-Hess ≥4** | 0.9541 | 0.4236 | 5.0736 | 0.0243 | 2.5963 | 1.1319 to 5.9554 |
| Vasospasm | 1.4595 | 0.4914 | 8.8211 | 0.0030 | 4.3038 | 1.6427 to 11.2758 |
| **Gender** | -0.1317 | 0.4665 | 0.07972 | 0.7777 | 0.8766 | 0.3513 to 2.1871 |
| Age | -0.00865 | 0.01780 | 0.2362 | 0.6269 | 0.9914 | 0.9574 to 1.0266 |
| **Ht-to-Hb>3.007** | 1.3505 | 0.5055 | 7.1371 | 0.0076 | 3.8594 | 1.4329 to 10.3949 |
| HTN | 0.04589 | 0.4735 | 0.009390 | 0.9228 | 1.0470 | 0.4139 to 2.6485 |
| Modified Fisher | -0.1534 | 0.2181 | 0.4944 | 0.4820 | 0.8578 | 0.5594 to 1.3154 |
| Anterior/Posterior | 0.2849 | 0.4375 | 0.4241 | 0.5149 | 1.3296 | 0.5641 to 3.1341 |
| Smoking | 0.1447 | 0.4498 | 0.1034 | 0.7478 | 1.1556 | 0.4786 to 2.7907 |
