# Supplementary material for: Hematocrit-to-Hemoglobin Ratio as a Novel Independent Predictor for In-Hospital Mortality and Delayed Cerebral Ischemia in Critically Ill Patients with Aneurysmal Subarachnoid Hemorrhage Requiring Neurosurgical or Endovascular Treatment: A Retrospective Analysis
Source: Neurocrit Care. 2025 Oct 24;44(2):527–40. doi: 10.1007/s12028-025-02395-x (PMC13053589; doi:10.1007/s12028-025-02395-x)
Supplement: Supplementary file 4 — Supplementary file4 (DOCX 1074 KB) [file 12028_2025_2395_MOESM4_ESM.docx]

**Supplementary material 4. Mortality analysis at days 30-, 60- and 90**

A

C

B

F

E

D

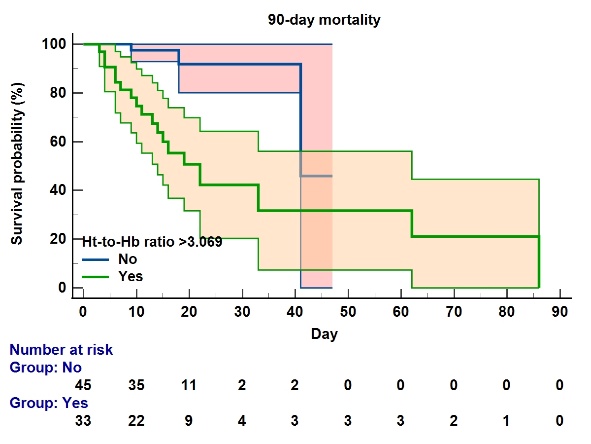

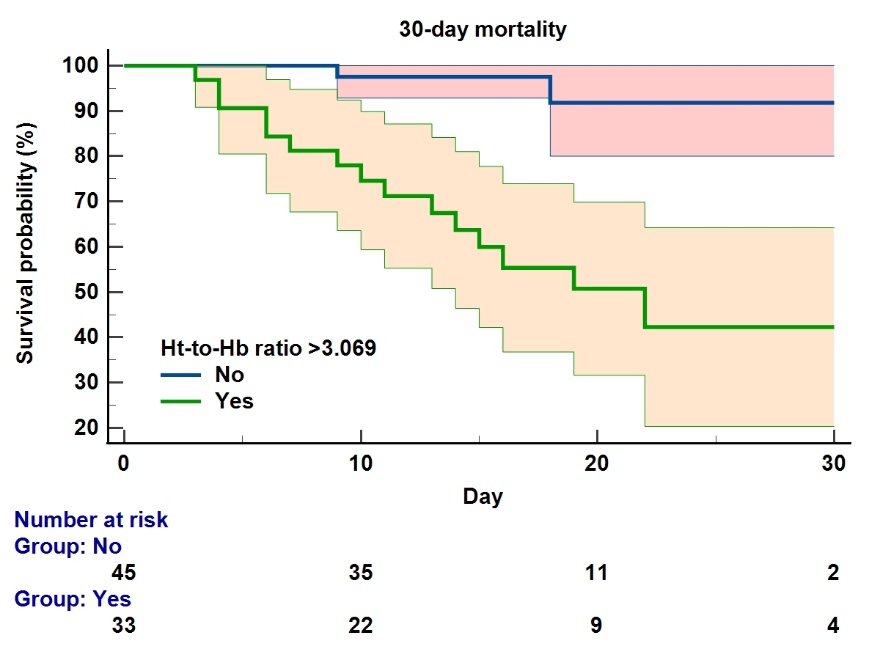

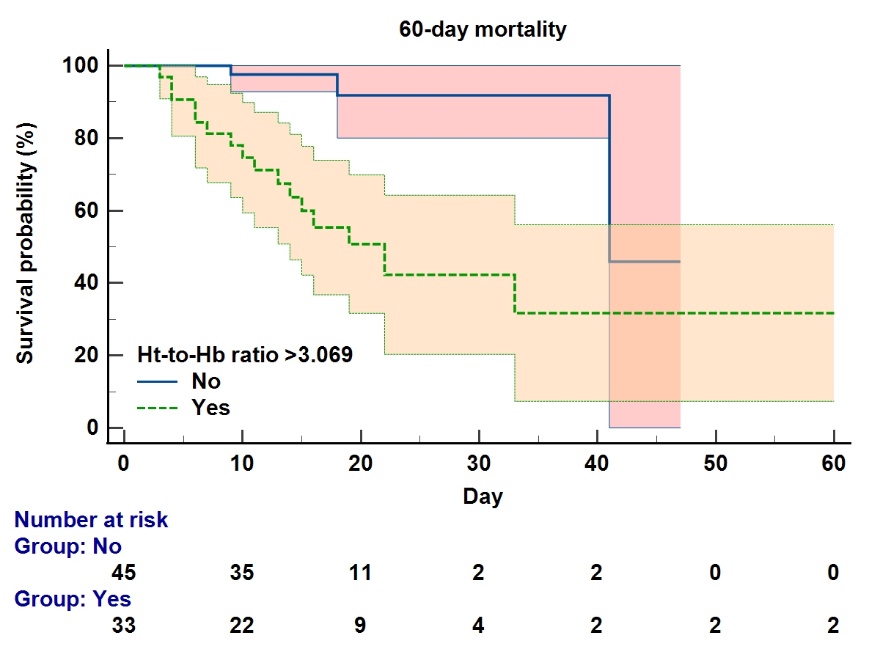

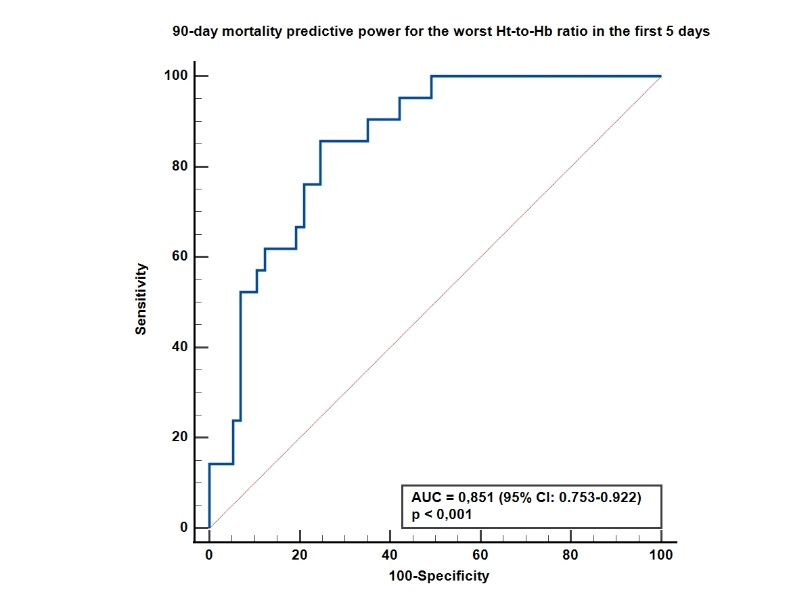

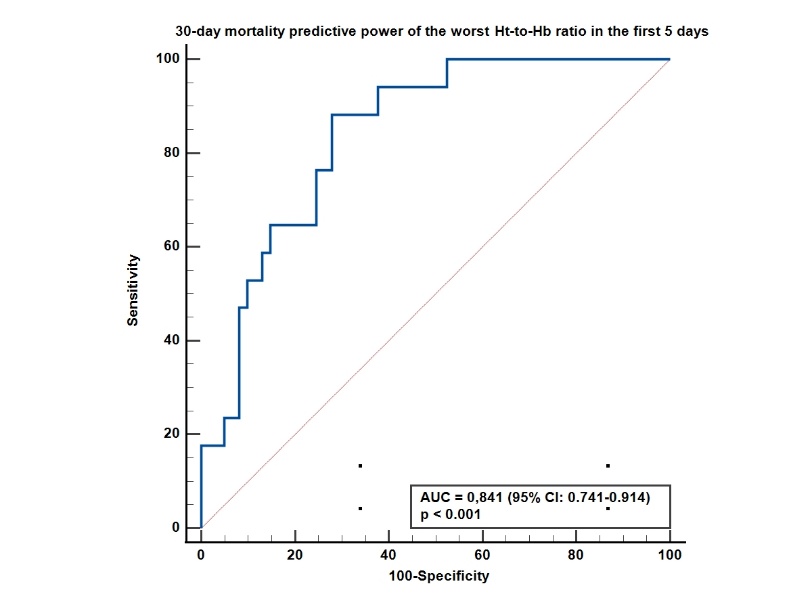

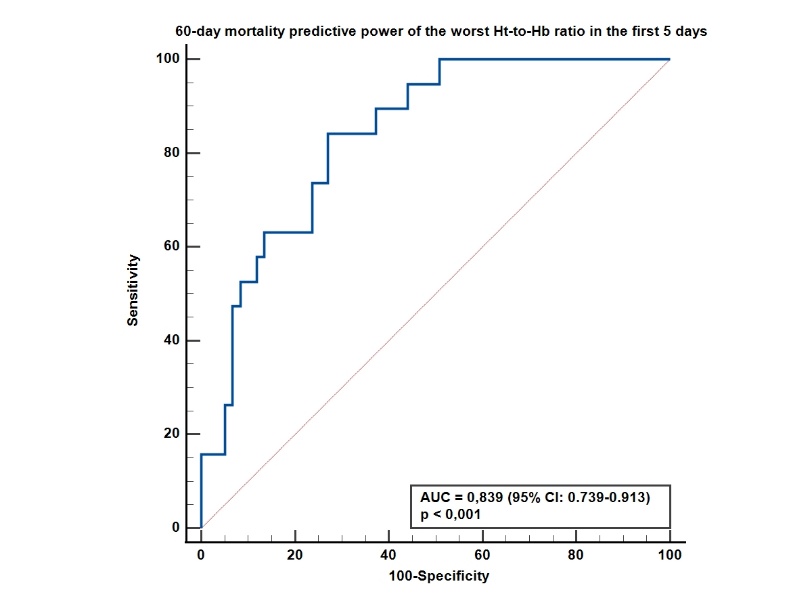


**Figure 1. Mortality analysis at 30-, 60- and 90-days. Figures 1A, 1B and 1C represent the Area Under the Receiver Operating Characteristics Analysis for 30-, 60- and 90-days mortality. The values of all three AUROCs varied marginally. Figures 1D, 1E and 1F represent the Kaplan-Meier Curves for survival probability at 30-, 60- and 90-days. Patients with a Ht-to-Hb ratio >3.069 had hazard ratios of 7.6 (95% CI: 2.85-20), 5.9 (95% CI: 2.35-14.82) and 5.8 (95% CI: 2.3-14.56) for 30-, 60- and 90-days mortality, respectively. The p value after the Log rank test was <0.001 for all three models. The mortality rates were as follows: 30-day: 17/78 (21.8%), 60-day: 19/78 24.35% and 90-day: 21/78 26.9%; 95% CI = 95% confidence interval; AUROC = area under the receiver operating characteristics**
